# A transdiagnostic examination of sex- and race and ethnicity-based mental health treatment disparities among publicly insured youth

**DOI:** 10.1017/S003329172510069X

**Published:** 2025-06-24

**Authors:** Erin C. Accurso, Megan E. Mikhail, Kate Duggento Cordell, Amanda E. Downey, Lonnie R. Snowden

**Affiliations:** 1 Department of Psychiatry and Behavioral Sciences, University of California, San Francisco, San Francisco, CA, USA; 2 Philip R. Lee Institute for Health Policy Studies, School of Medicine, University of California, San Francisco, San Francisco, CA, USA; 3 Department of Psychology, Michigan State University, East Lansing, MI, USA; 4 Mental Health Data Alliance, Folsom, CA, USA; 5 Center for Innovation in Population Health, University of Kentucky, Lexington, KY, USA; 6 Social Policy Institute, San Diego State University, San Diego, CA, USA; 7 Department of Pediatrics, University of California, San Francisco, San Francisco, CA, USA; 8 School of Public Health, University of California, Berkeley, CA, USA

**Keywords:** eating disorders, anxiety disorders, mood disorders, psychotic disorders, Medicaid, health disparities, health services, adolescent

## Abstract

**Background:**

Low-income, publicly insured youth face numerous barriers to adequate mental health care, which may be compounded for those with multiple marginalized identities. However, no research has examined how identity and diagnosis may interact to shape the treatment experiences of under-resourced youth with psychiatric conditions. Applying an intersectional lens to treatment disparities is essential for developing targeted interventions to promote equitable care.

**Methods:**

Analyses included youth ages 7–18 with eating disorders (EDs; *n* = 3,311), mood/anxiety disorders (*n* = 3,219), or psychotic disorders (*n* = 3,035) enrolled in California Medicaid. Using state billing records, we examined sex- and race and ethnicity-based disparities in receipt of core services – outpatient therapy, outpatient medical care, and inpatient treatment – in the first year after diagnosis and potential differences across diagnostic groups.

**Results:**

Many youth (50.7% across diagnoses) received no outpatient therapy, and youth with EDs were least likely to receive these services. Youth of color received fewer days of outpatient therapy than White youth, and Latinx youth received fewer therapy and medical services across outpatient and inpatient contexts. Sex- and race and ethnicity-based disparities were especially pronounced for youth with EDs, with particularly low levels of service receipt among boys and Latinx youth with EDs.

**Conclusions:**

Results raise concerns for unmet treatment needs among publicly insured youth, which are exacerbated for youth with multiple marginalized identities and those who do not conform to historical stereotypes of affected individuals (e.g., low-income boys of color with EDs). Targeted efforts are needed to ensure equitable care.

Socioeconomically disadvantaged youth are at increased risk of psychiatric conditions due to the considerable stressors associated with living in under-resourced contexts (e.g., fewer material resources, increased exposure to crime and trauma, family relational stress; Reiss, 2013; Ridley, Rao, Schilbach, & Patel, 2020; Yoshikawa, Aber, & Beardslee, 2012). Poverty is associated with mental health concerns transdiagnostically, including mood/anxiety disorders (Najman et al., 2010; Ridley et al., 2020), eating disorders (EDs; Burnette, Burt, & Klump, 2024), and psychotic disorders (Karcher, Schiffman, & Barch, 2021; Wicks, Hjern, Gunnell, Lewis, & Dalman, 2005). However, economically disadvantaged youth face significant barriers to receiving adequate mental health care (Castro-Ramirez et al., 2021; DeCarlo Santiago et al., 2013). The negative ramifications of insufficient mental health treatment are wide-ranging and long-lasting. Poorer mental health in adolescence is associated with lower academic achievement (Brännlund, Strandh, & Nilsson, 2017), higher adult unemployment (Clayborne, Varin, & Colman, 2019), worse adult social functioning (Chen et al., 2006), and increased mortality (Hoang, Goldacre, & James, 2014; Simon, Stewart, Yarborough et al., 2018). Addressing unmet treatment needs and disparities in access to care is therefore critical to advance health equity.

While youth from under-resourced backgrounds are less likely to receive mental health treatment overall, barriers to care may be compounded by biases, stigma, and other systemic factors for youth with additional marginalized identities. In particular, youth of color may be less likely to be identified as having a mental illness, encounter other forms of prejudice/bias within care systems, and/or experience increased stigma within their cultural context, all which could contribute to reduced treatment engagement relative to other low-income youth (Castro-Ramirez et al., 2021; Metzger et al., 2023; Misra et al., 2021). The impact of identity factors on care may also depend on the stereotypes associated with particular mental health diagnoses. For example, boys with EDs may be overlooked due to the perception that these conditions predominately impact girls (Gorrell & Murray, 2019) and some affective disorders tend to be under-recognized in Black individuals (Akinhanmi et al., 2018). Intersectional approaches to understanding health inequities are crucial to shed light on how social identities (e.g., race, sex, and socioeconomic status) may interact with health factors (e.g., diagnosis) to shape treatment experiences.

Despite the potential influence of intersectionality on mental health treatment disparities, little research has explored how demographic factors affect care receipt among under-resourced youth or whether these effects differ across diagnoses. Recent research among youth with EDs suggests under-resourced male and Latinx youth may be particularly likely to experience gaps in care (Mikhail, Cordell, Downey, Snowden, & Accurso, 2025; Moreno, Buckelew, Accurso, & Raymond-Flesch, 2023), but it is unclear whether these findings are more broadly applicable across diagnoses. Identifying the characteristics of youth at greatest risk for unmet treatment needs – and the extent to which these differ across diagnostic populations – is key to developing targeted, effective interventions to address inequities.

Within the United States (US), low-income youth and families are eligible for public health insurance through Medicaid, which provides physical and behavioral health care coverage for over 32 million children and adolescents (Centers for Medicare and Medicaid Services [CMS], 2017). Medicaid-insured youth are racially and ethnically diverse, with a majority identifying as individuals of color (CMS, 2020). Medicaid data can therefore provide uniquely valuable insight into intersectional factors that may impact the treatment experiences of under-resourced youth with psychiatric diagnoses. Given Medicaid’s expansive scope, addressing disparities within the program could also yield far-reaching benefits for marginalized youth.

In the current study, we leveraged Medicaid claims data to understand how receipt of psychological therapy, medical care, and more intensive inpatient treatment differed across demographic and diagnostic factors in under-resourced youth with mental health diagnoses. We focused on three categories of diagnoses: mood/anxiety disorders, EDs, and psychotic disorders. Mood/anxiety disorders are extremely common, with a combined lifetime prevalence >30% in adolescents (Kessler, Petukhova, Sampson, Zaslavsky, & Wittchen, 2012), contributing significantly to the public health burden (Wittchen, 2012). While EDs and psychotic disorders are rarer (prevalence rates of ~10% and <5%, respectively; Calkins et al., 2014; Herpertz-Dahlmann, 2015), they are associated with high morbidity and frequent need for intensive services. Although treatment experiences may overlap across these disorders in some ways (e.g., psychotherapy as a core intervention; Datta, Matheson, Citron, Van Wye, & Lock, 2023; Müller, Laier, & Bechdolf, 2014; Verdeli, Mufson, Lee, & Keith, 2006), they also differ in their associated stereotypes with respect to income, race and ethnicity, and sex (Gorrell & Murray, 2019; Mikhail & Klump, 2021; Schwartz & Blankenship, 2014) and possible barriers to care (Causier, Waite, Sivarajah, & Knight, 2024; Innes, Clough, & Casey, 2017; Mechanic, 2007). Treatment for mood/anxiety disorders is also often perceived as less specialized than treatment for EDs and psychotic disorders, which may contribute to more trained providers and increased accessibility. Examining patterns of treatment disparities across these diagnoses can therefore illuminate which inequities are transdiagnostic and which may be shaped by disorder-specific factors. We hypothesized that we would find relatively high levels of unmet treatment needs across diagnoses, with added treatment disparities for youth of color. We also hypothesized that youth with EDs would show greater sex- and race-based treatment inequities than youth with other psychiatric disorders due to pervasive stereotypes that EDs predominately impact White girls.

## Methods

### Participants

Participants with EDs, mood/anxiety disorders, and psychotic disorders were drawn from the full population of California Medicaid beneficiaries ages 7–18 with at least one service episode between January 1, 2014 and December 31, 2016 (*N* = 4,819,221 unique beneficiaries; Accurso, Cordell, Guydish, & Snowden, 2024). Although these disorders typically emerge in adolescence, they can develop earlier, so we included youth as young as 7 to capture a fuller range of presentations. California’s Medicaid program (Medi-Cal) is the largest of any state, serving youth across 58 counties diverse in size, urbanicity, socioeconomic resources, and provider capacity. In addition to its large member base, Medi-Cal has the potential to be particularly informative because it is administered independently by each county in California, creating a variety of service environments that reflect the diversity found across the US within a single state.

The parent study from which these data were drawn focused primarily on the treatment of publicly insured youth with EDs (Accurso et al., 2024). We therefore initially identified all youth with an ICD-9 or ICD-10 ED billing diagnosis in the population (*n* = 8,075). Simple random sampling procedures were then implemented in SAS to extract comparison sets of youth with a mood/anxiety (*n* = 8,000) or psychotic disorder (*n* = 8,000) billing diagnosis (see Supplemental Material for additional information regarding definitions and classification of youth in each group, including for youth with diagnoses across multiple categories). Random samples of youth with mood/anxiety and psychotic disorders were utilized rather than the full populations of these youth due to California Department of Health Care Services (DHCS) protocols that restrict data release to the “minimum necessary” to address research questions.

Primary analyses evaluated services received in the first year after known diagnosis among youth continuously enrolled in Medi-Cal across that period (EDs: *n* = 3,311, 34.6%; mood/anxiety disorders: *n* = 3,219, 33.7%; psychotic disorders: *n* = 3,035, 31.7%) to ensure all relevant services were captured and evaluated over the same timeframe for all youth. Descriptive data are also provided for the second year after known diagnosis for youth who had two full years of Medi-Cal enrollment following diagnosis (see Supplementary Table S1).

### Measures

#### Service utilization

Service utilization data were drawn from billing claims, which included the date(s), type, code, and billing diagnoses for each service received. We focused on three types of services: outpatient psychotherapy, outpatient medical care, and inpatient admissions. Examining outpatient therapy was important because this represents a core intervention for youth mental health concerns. Analyses of outpatient medical care allowed us to examine the medical management of psychiatric diagnoses (e.g., medical care supporting overall health, specific treatment for medical sequelae of mental health diagnoses, and medication side effects), while analyses of inpatient treatment allowed us to examine the use of intensive, high-cost services for the most severely affected youth.

*Outpatient psychotherapy* included services billed with Current Procedural Terminology (CPT) codes for individual or family therapy (90832–90834, 90836–90840, 90846, 90847, 90875, 90876, and H0032). *Outpatient medical* care included all outpatient medical services not captured in the category of mental health services described above. *Inpatient* services included all services with an inpatient claim type. Supplemental analyses that separately examined inpatient admissions with primarily (≥50%) mental health claims and primarily medical claims are included in Table S2 and Figures S1 and S2 in the Supplementary Material.

#### Demographic variables

Demographic variables included sex, race and ethnicity, and earliest age of known diagnosis within the sampling timeframe (calculated from birth month/year and the date of the first claims record in the dataset with an applicable diagnosis). Race and ethnicity were collected as a single variable by DHCS, which were grouped into the following exclusive categories: Asian/Pacific Islander, Black/African-American, Hispanic/Latinx, White, and other/unknown. Participants in the other/unknown category included those whose race and ethnicity were reported as “other,” “unknown,” or “not reported,” as well as participants who identified as “Alaskan Native or American Indian” (*n* = 31) due to small sample size.

### Statistical analyses

We first examined descriptive patterns of service use to understand receipt of core treatment components across the overall population, identify potential gaps in care (e.g., number of youth receiving no outpatient therapy), and assess overall differences in treatment experiences across diagnoses. We then examined how youth demographic and diagnostic characteristics predicted service receipt when included together in the same model. These analyses used Poisson regression with robust error variance (Zou, 2004) for categorical outcomes (odds of receiving any outpatient therapy and odds of an inpatient admission) and negative binomial regression for count outcomes (number of days of outpatient therapy, outpatient medical care, and inpatient treatment). We examined both the receipt of any care and the number of days of care for outpatient therapy and inpatient admissions because different factors may impact disparities in initial care receipt and care duration/retention. Conversely, we only examined a number of days of outpatient medical care because almost all youth (96.7% across the full sample) received at least some outpatient medical services. Each service type was analyzed separately because services are not interchangeable (e.g., medical care helps stabilize physical health but cannot replace therapy for addressing underlying psychopathology). However, supplemental analyses covarying other service types showed a similar pattern of effects (see Supplementary Table S3). Finally, we performed interaction analyses to examine whether sex- and race and ethnicity-based disparities in treatment receipt differed across diagnoses.

## Results

### Service receipt

#### Outpatient services

Across diagnoses, a striking number of youth received no outpatient therapy despite having a known diagnosis (50.7% across the full sample; see Table 1 for descriptive statistics across services), with significant differences between diagnoses. Youth with EDs were significantly less likely to receive any outpatient therapy than youth with mood/anxiety disorders (38.6% vs. 51.4%, *p* < .001), who were less likely to receive outpatient therapy than youth with psychotic disorders (58.8%; *p* < .001). Diagnostic disparities in therapy receipt were particularly pronounced for services billed as family therapy (EDs = 29.2%, mood/anxiety disorders = 41.8%, psychotic disorders = 54.6%). When youth did receive outpatient therapy, the frequency was low across diagnoses, though slightly higher for youth with psychotic disorders (*M* = 7.5 days) relative to youth with EDs (*M* = 5.7 days; *p* < .001 relative to psychotic disorders) and mood/anxiety disorders (*M* = 5.1 days; *p* = .021 relative to EDs). While youth with EDs were least likely to receive outpatient therapy, they received the most outpatient medical care (*M* = 15.9 days vs. 12.6 days for psychotic disorders and 9.1 days for mood/anxiety disorders; *p*s < .001).

**Table 1.** Descriptive statistics for participant demographics and service use in the first year after known diagnosis

Demographic variables	Eating disorders (ED)		Mood and anxiety disorders (MAD)		Psychotic disorders (PD)		Group comparison
	N	%	N	%	N	%	
All	3,311	–	3,219	–	3,035	–	–
Sex							
Female	2,270	68.6	1,756	54.6	1,565	51.6	PD < MAD < ED
Male	1,041	31.4	1,463	45.4	1,470	48.4	–
Race and ethnicity							
Asian/Pacific Islander	164	5.0	121	3.8	96	3.2	MAD = PD < ED
Black/African American	130	3.9	229	7.1	257	8.5	ED < MAD < PD
Latinx	1,842	55.6	1,566	48.6	1,112	36.6	PD < MAD < ED
Other/Unknown	436	13.2	368	11.4	554	18.3	MAD < ED < PD
White	739	22.3	935	29.0	1,016	33.5	ED < MAD < PD
Language							
English	1,700	51.3	2,051	63.7	2,126	70.0	ED < MAD < PD
Other	1,611	48.7	1,168	36.3	909	30.0	–
	Median	Mean (SD)	Median	Mean (SD)	Median	Mean (SD)	
Age at known diagnosis	13.6	12.8 (2.9)	13.2	12.8 (2.9)	14.2	13.6 (2.6)	ED = MAD < PD
Service use	N (%)	Mean (SD)	N (%)	Mean (SD)	N (%)	Mean (SD)	
Any outpatient therapy	1,277 (38.6)	5.7 (6.8)	1,655 (51.4)	5.1 (6.5)	1,785 (58.8)	7.5 (9.5)	N: ED < MAD < PD Days: MAD < ED < PD
Family therapy	966 (29.2)	4.8 (6.1)	1,344 (41.8)	4.6 (6.1)	1,657 (54.6)	7.4 (9.6)	N: ED < MAD < PD Days: ED = MAD < PD
Individual therapy	463 (14.0)	5.7 (6.6)	380 (11.8)	6.1 (6.8)	217 (7.1)	4.5 (5.0)	N: PD < MAD < ED Days: PD < ED = MAD
Outpatient medical services	3,258 (98.4)	15.9 (24.2)	3,054 (94.9)	9.1 (11.6)	2,941 (96.9)	12.6 (14.9)	N: MAD < PD < ED Days: MAD < PD < ED
Inpatient admissions	622 (18.8)	30.1 (76.8)	336 (10.4)	9.5 (23.7)	1,508 (49.7)	11.9 (17.7)	N: MAD < ED < PD Days: MAD < PD < ED
MH admissions	492 (14.9)	20.4 (49.2)	269 (8.4)	9.2 (23.2)	1,474 (48.6)	11.7 (17.2)	N: MAD < ED < PD Days: MAD < PD < ED
Medical admissions	151 (4.6)	57.6 (124.6)	79 (2.5)	8.8 (23.2)	67 (2.2)	9.7 (22.3)	N: MAD = PD < ED Days: MAD = PD < ED

*Note.* ED = eating disorder; MAD = mood/anxiety disorder; PD = psychotic disorder; MH = mental health; SD = standard deviation; N = number of participants receiving a given service; Days = number of days on which a service was received. Individual therapy was identified based on the following Current Procedural Terminology (CPT) codes: 90832–90834, 90836–90840, 90875, and 90876. Family therapy was identified using CPT codes 90846, 90847, and H0032. Means and standard deviations for service use categories are provided for participants who received at least one day of that service. Group comparisons were evaluated using chi-squared tests for categorical variables and *t*-tests for continuous variables at a significance level of *p* < .05. Dashes indicate that a value is not applicable (or is implied by a previous comparison).

#### Inpatient services

Youth with psychotic disorders were most likely to experience a hospitalization in the first year after a known diagnosis (49.7%), followed by youth with EDs (18.8%; *p* < .001 relative to youth with psychotic disorders) and youth with mood/anxiety disorders (10.4%; *p* < .001 relative to youth with EDs). Concerningly, large proportions of youth who were hospitalized received no outpatient therapy at all either before or after hospitalization, with this percentage greatest for youth with EDs (45.5% of admitted youth with no outpatient therapy), followed by youth with mood/anxiety disorders (38.7%, *p* = .042 relative to youth with EDs), then youth with psychotic disorders (31.6%, *p* = .012 relative to youth with mood/anxiety disorders). Once hospitalized, youth with EDs had significantly longer admissions (*M* = 30.1 days) than youth with psychotic disorders (*M* = 11.9 days) and youth with mood/anxiety disorders (*M* = 9.5 days) (eating vs. psychotic disorders: *p* < .001; psychotic vs. mood/anxiety disorders: *p* = .038). Primarily medical admissions for youth with EDs tended to be particularly long (*M* = 57.6 days).

### Sex differences in service receipt

#### Full sample

Across the full sample, females received fewer days of outpatient medical care than males (IRR = .92, 95% CI [.88, .95]). Conversely, females were more likely than males to receive any outpatient therapy (IRR = 1.09, 95% CI [1.04, 1.14]) and experience an inpatient admission (IRR = 1.40, 95% CI [1.31, 1.50]) (see Table 2). Days of outpatient therapy and length of inpatient admissions did not significantly differ across sexes.

**Table 2.** Effects of demographic and diagnostic variables in predicting service receipt in the first year after known diagnosis across the full sample

	Any outpatient therapy services	Days outpatient therapy services	Days outpatient medical services	Any inpatient admission	Days Inpatient
	IRR (95% CI)	IRR (95% CI)	IRR (95% CI)	IRR (95% CI)	IRR (95% CI)
Diagnostic group (Ref = ED)					
Mood/anxiety disorder	**1.34 (1.27, 1.42)** ***	**.89 (.82, .95)** **	**.58 (.55, .60)** ***	**.59 (.52, .66)** ***	**.36 (.31, .42)** ***
Psychotic disorder	**1.51 (1.43, 1.59)** ***	**1.27 (1.19, 1.37)** ***	**.78 (.75, .82)** ***	**2.56 (2.36, 2.77)** ***	**.49 (.44, .55)** ***
Sex (Ref = Male)					
Female	**1.09 (1.04, 1.14)** ***	1.02 (.96, 1.09)	**.92 (.88, .95)** ***	**1.40 (1.31, 1.50)** ***	1.01 (.92, 1.11)
Race and ethnicity (Ref = White)					
Asian/Pacific Islander	.97 (.87, 1.08)	**.79 (.68, .92)** **	**.84 (.76, .94)** **	.88 (.74, 1.04)	1.12 (.90, 1.41)
Black	.95 (.88, 1.04)	**.79 (.70, .89)** ***	**.86 (.79, .94)** **	.81 (.70, .93) **	.92 (.76, 1.11)
Latinx	**.91 (.87, .95)** ***	**.80 (.75, .86)** ***	**.76 (.73, .80)** ***	**.82 (.76, .88)** ***	**.72 (.65, .79)** ***
Other/Unknown	.96 (.90, 1.02)	**.86 (.78, .94)** **	**1.11 (1.04, 1.18)** **	.95 (.86, 1.04)	**1.68 (1.47, 1.92)** ***
Age at first known diagnosis	**1.01 (1.01, 1.02)** ***	**.98 (.97, .995)** **	**.97 (.96, .97)** ***	**1.11 (1.10, 1.13)** ***	**.93 (.92, .95)** ***

*Note.* ED = eating disorder; IRR = incidence rate ratio. The number of service days was analyzed for participants who received at least one instance of a given service type. Receipt of any outpatient medical services was not examined as an outcome because almost all youth received at least some outpatient medical care. *p < .05; **p < .01; ***p < .001.

#### Differences across diagnoses

**Figure 1.**
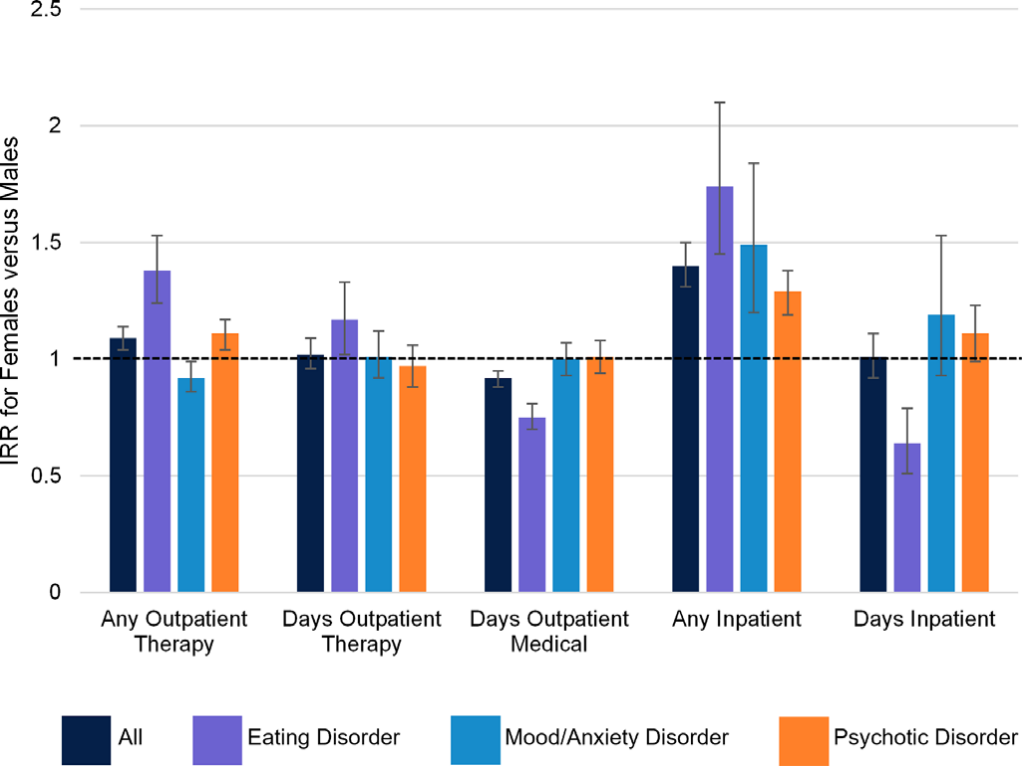
*Sex differences in services received by diagnosis.* IRR = incidence rate ratio. Error bars represent 95% confidence intervals and the dotted line represents an IRR of 1 (indicating no difference between females and males).

Diagnosis × sex interactions suggested sex disparities in treatment receipt tended to be greater for youth with EDs relative to youth with other disorders (see Table 3 and Figure 1). Among youth with EDs, females were significantly more likely than males to receive any outpatient therapy (IRR = 1.38, 95% CI [1.24, 1.53]) and received more days of outpatient therapy (IRR = 1.17, 95% CI [1.02, 1.33]). Females were also more likely to be hospitalized (IRR = 1.74, 95% CI [1.45, 2.10]). However, females with EDs received significantly less outpatient medical care than males (IRR = .75, 95% CI [.70, .81]) and had significantly shorter hospitalizations once admitted (IRR = .64, 95% CI [.51, .79]). Conversely, sex differences in service receipt tended to be smaller or non-significant in youth with mood/anxiety and psychotic disorders, with the exception that females were more likely than males to be hospitalized across all three diagnostic categories (though this sex disparity was significantly smaller for youth with psychotic disorders than youth with EDs).

**Table 3.** Interactions between demographic and diagnostic variables in predicting service receipt in the first year after known diagnosis

	Any outpatient therapy services	Days outpatient therapy services	Days outpatient medical services	Any inpatient admission	Days inpatient
	IRR (95% CI)	IRR (95% CI)	IRR (95% CI)	IRR (95% CI)	IRR (95% CI)
Diagnostic group (Ref = ED)					
Mood/anxiety disorder (MAD)	**1.55 (1.36, 1.78)** ***	.92 (.77, 1.10)	**.45 (.41, .51)** ***	**.51 (.38, .68)** ***	**.27 (.19, .38)** ***
Psychotic disorder (PD)	**1.66 (1.45, 1.89)** ***	**1.48 (1.25, 1.75)** ***	**.66 (.59, .73)** ***	**2.50 (2.04, 3.08)** ***	**.44 (.34, .58)** ***
Sex (Ref = Male)					
Female	**1.38 (1.24, 1.53)** ***	**1.17 (1.02, 1.33)** *	**.75 (.70, .81)** ***	**1.74 (1.45, 2.10)** ***	**.64 (.51, .79)** ***
Race and Ethnicity (Ref = White)					
Asian/Pacific Islander	.98 (.81, 1.18)	.86 (.67, 1.11)	**.78 (.66, .91)** **	**.67 (.48, .94)** *	**1.66 (1.12, 2.45)** *
Black	**.77 (.60, .99)** *	.93 (.68, 1.26)	.97 (.81, 1.15)	.81 (.57, 1.14)	**1.69 (1.11, 2.56)** *
Latinx	**.82 (.74, .91)** ***	**.78 (.69, .89)** ***	**.72 (.66, .78)** ***	**.54 (.47, .64)** ***	**.68 (.56, .82)** ***
Other/Unknown	.91 (.79, 1.05)	**.80 (.66, .96)** *	**1.17 (1.05, 1.31)** **	.98 (.80, 1.19)	**3.66 (2.85, 4.70)** ***
Age at first known diagnosis	**1.01 (1.01, 1.02)** **	**.98 (.97, .99)** **	**.97 (.96, .97)** ***	**1.11 (1.10, 1.12)** ***	**.95 (.93, .96)** ***
*Model interaction terms*					
Diagnosis × Sex					
MAD × sex	**.67 (.59, .76)** ***	.87 (.74, 1.02)	**1.32 (1.20, 1.46)** ***	.85 (.64, 1.13)	**1.87 (1.34, 2.61)** ***
PD × sex	**.80 (.71, .90)** ***	**.83 (.71, .97)** *	**1.33 (1.21, 1.47)** ***	**.74 (.60, .90)** **	**1.74 (1.36, 2.22)** ***
Diagnosis × race and ethnicity					
MAD × Asian/Pacific Islander	.98 (.75, 1.28)	.89 (.61, 1.29)	1.25 (.98, 1.59)	1.43 (.77, 2.65)	1.00 (.49, 2.03)
MAD × Black	**1.48 (1.12, 1.96)** **	.88 (.61, 1.27)	.84 (.67, 1.05)	1.12 (.65, 1.94)	.57 (.30, 1.08)
MAD × Latinx	**1.19 (1.05, 1.35)** **	1.10 (.93, 1.31)	1.12 (1.00, 1.25)	**1.51 (1.14, 2.02)** **	1.20 (.86, 1.69)
MAD × other/unknown	1.08 (.90, 1.30)	1.23 (.96, 1.57)	1.06 (.90, 1.24)	**1.56 (1.09, 2.23)** *	**.39 (.25, .59)** ***
PD × Asian/Pacific Islander	.96 (.74, 1.24)	.86 (.59, 1.24)	1.02 (.79, 1.32)	1.43 (.97, 2.10)	**.50 (.30, .81)** **
PD × Black	1.16 (.88, 1.54)	.80 (.56, 1.14)	.88 (.71, 1.10)	.99 (.68, 1.45)	**.46 (.28, .73)** **
PD × Latinx	1.13 (1.00, 1.28)	.98 (.83, 1.16)	1.06 (.95, 1.19)	**1.82 (1.52, 2.17)** ***	1.03 (.82, 1.29)
PD × other/unknown	1.07 (.91, 1.27)	1.03 (.82, 1.29)	**.84 (.72, .97)** *	.87 (.70, 1.10)	**.27 (.20, .37)** ***

*Note*: ED = eating disorder; MAD = mood/anxiety disorder; PD = psychotic disorder; IRR = incidence rate ratio. The number of service days was analyzed for participants who received at least one instance of a given service type. Receipt of any outpatient medical services was not examined as an outcome because almost all youth received at least some outpatient medical care. *p < .05; **p < .01; ***p < .001.

### Racial and ethnic differences in service receipt

#### Full sample

In the full sample, race and ethnicity-based treatment disparities were particularly pronounced with respect to number of days of outpatient therapy (see Table 2). All youth of color, including Asian/Pacific Islander (IRR = .79, 95% CI [.68, .92]), Black (IRR = .79, 95% CI [.70, .89]), Latinx (IRR = .80, 95% CI [.75, .86]), and youth of other/unknown race (IRR = .86, 95% CI [.78, .94]) received fewer days of outpatient therapy than White youth.

Notably, Latinx youth also received fewer services than White youth across all other service types (any outpatient therapy: IRR = .91, 95% CI [.87, .95]; days outpatient medical care: IRR = .76, 95% CI [.73, .80]; any inpatient admission: IRR = .82, 95% CI [.76, .88]; days inpatient: IRR = .72, 95% CI [.65, .79]), a pattern which continued into the second year after the known diagnosis (see Supplementary Table S4). Black (IRR = .86, 95% CI [.79, .94]) and Asian/Pacific Islander youth (IRR = .84, 95% CI [.76, .94]) had fewer days of outpatient medical care than White youth and Black youth were also less likely to be hospitalized (IRR = .81, 95% CI [.70, .93]). Interestingly, youth of other/unknown race received more days of outpatient medical care (IRR = 1.11, 95% CI [1.04, 1.18]) and had longer inpatient admissions (IRR = 1.68, 95% CI [1.47, 1.92]) relative to White youth.

#### Differences across diagnoses

**Figure 2.**
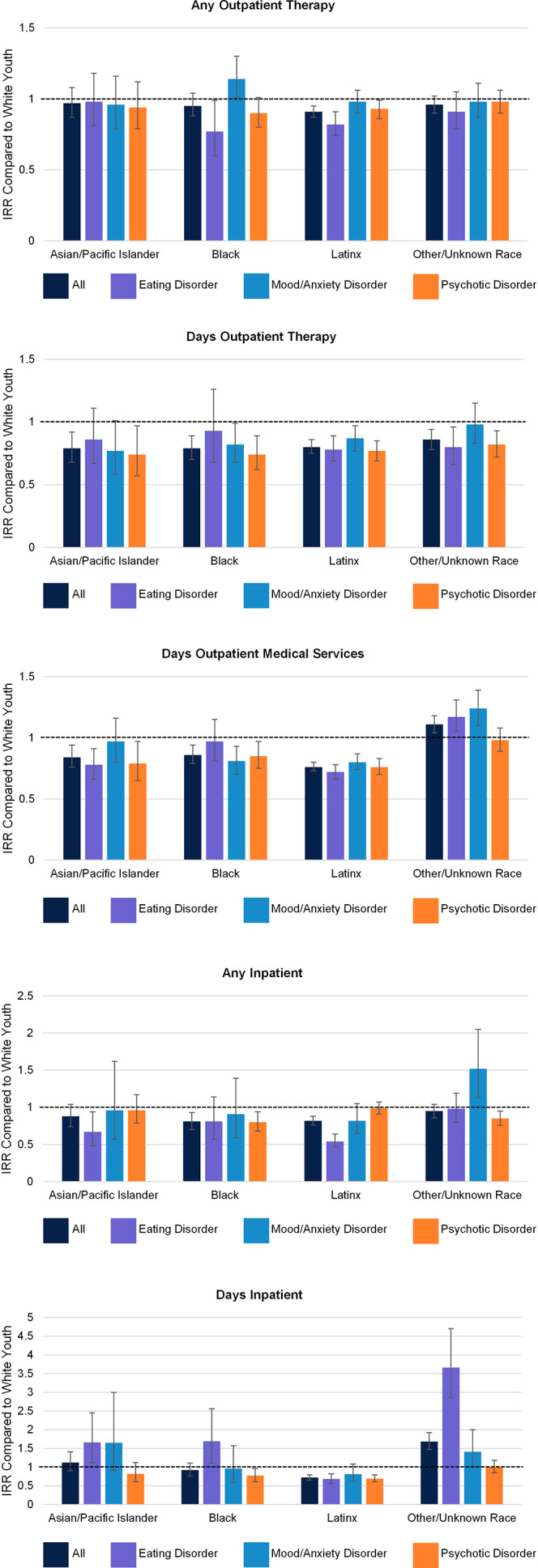
*Racial and ethnic differences in services received by diagnosis.* IRR = incidence rate ratio. Error bars represent 95% confidence intervals and the dotted line represents an IRR of 1 (indicating no difference with White youth).

When significant interaction effects were present, they again tended to suggest greater racial disparities in treatment receipt for youth with EDs relative to youth with other psychiatric diagnoses (see Table 3 and Figure 2). Among youth with EDs, both Black (IRR = .77, 95% CI [.60, .99]) and Latinx (IRR = .82, 95% CI [.74, .91]) youth were significantly less likely to receive any outpatient therapy than White youth. Associations between Black or Latinx identity and receipt of outpatient therapy were weaker for youth with mood/anxiety disorders (*p*s < .05 for race and ethnicity × diagnosis interactions; see Table 3), with Latinx (IRR = .98, 95% CI [.90, 1.06]) and Black (IRR = 1.14, 95% CI [1.01, 1.30]) youth with mood/anxiety disorders at least as likely to receive outpatient therapy as White youth. Conversely, except for Latinx youth, youth of color with EDs tended to have longer hospitalizations than White youth (Asian/Pacific Islander: IRR = 1.66, 95% CI [1.12, 2.45]; Black: IRR = 1.69, 95% CI [1.11, 2.56]; other/unknown race: IRR = 3.66, 95% CI [2.85, 4.70]). This pattern was attenuated for youth with psychotic disorders (and to a lesser extent youth with mood/anxiety disorders), who showed fewer differences in length of hospitalization by race and ethnicity.

#### Exploratory analyses of preferred language

We wondered whether racial disparities in service receipt might be attributable in part to language barriers; we therefore conducted supplemental models with preferred language in place of race and ethnicity (see Table S5 and Figure S3 in the Supplementary Material). Interestingly, disparities were somewhat smaller across preferred language in the full sample, with youth who preferred a language other than English receiving outpatient therapy at similar rates and for a similar number of days as youth with English as a preferred language. However, youth with EDs generally showed stronger disparities across language than youth with other diagnoses. In particular, youth with EDs who preferred a language other than English were significantly less likely to receive outpatient therapy than English-speaking youth (IRR = .90, 95% CI [.82, .98]).

## Discussion

This is the first study to examine intersectional disparities in service receipt across demographic and diagnostic characteristics among publicly insured youth with psychiatric conditions. Analyses revealed high hospitalization rates in the first year following diagnosis for youth with psychotic disorders (≈50%) and EDs (≈20%). Alarmingly, across diagnoses, many youth received no outpatient psychotherapy even when experiencing severe symptoms requiring inpatient admission. However, not all youth experienced gaps in care to the same degree. While the mean number of outpatient therapy sessions was relatively low for all youth, youth of color received even fewer sessions than White youth. Treatment disparities were particularly striking for Latinx youth, who received the least care across service types. Youth with EDs were less likely than youth with other psychiatric diagnoses to receive any outpatient therapy, despite the fact that outpatient therapy is the primary evidence-based intervention for adolescents with EDs (Datta et al., 2023). Inequities in care were compounded for youth who had an ED *and* an identity that did not fit common ED stereotypes, including boys and youth of color. Overall, these data provide novel insight into treatment disparities among an already vulnerable population, identifying key points for intervention to improve health equity.

The high percentage of youth who received no outpatient therapy across the full sample suggests significant unmet treatment needs in this population transdiagnostically. Lack of treatment may have a profound impact on clinical course, costs, and outcomes, with research suggesting that outpatient therapy significantly reduces hospitalizations for psychotic disorders (Marcus, Chuang, Ng-Mak, & Olfson, 2017) and EDs (Lock et al., 2016; Mikhail et al., in press). When youth did receive care, the average number of sessions provided (5–7) was well below that of even the most streamlined, manualized protocols (e.g., 13 in the adolescent Unified Protocol for mood/anxiety disorders, 20 in Family-Based Treatment for EDs; Ehrenreich, Goldstein, Wright, & Barlow, 2009; Gorrell, Loeb, & Le Grange, 2019) and empirically derived recommendations (e.g., ≥25 sessions for cognitive-behavioral therapy for psychosis; Lincoln, Jung, Wiesjahn, & Schlier, 2016). This is despite the fact that additional time may be needed to establish trust and understand youths’ sociocultural context in under-resourced settings.

Treatment disparities were compounded among youth of color, and Latinx youth in particular, placing these youth at elevated risk for undertreatment and poor outcomes. Systems-based (e.g., biases in treatment referral) and youth/family-based (e.g., stigma) factors that delay treatment may also contribute to increased severity upon presentation to care, leading to increased need for medical intervention and longer hospitalizations. While prior research has suggested that youth of color are less likely to receive treatment for mental health conditions (Castro-Ramirez et al., 2021), our study indicates that these inequities persist even among low-income, publicly insured youth. In other words, poor youth of color are doubly disadvantaged in receiving appropriate treatment due to additive effects of race and ethnicity and socioeconomic status. Our findings are also notable because they were observed among youth with a recognized mental health diagnosis. While bias impacts the detection of psychiatric conditions (Garb, 2021; Liang, Matheson, & Douglas, 2016), these data indicate that health disparities persist even after youth are identified and formally diagnosed. Most striking was that across diagnoses, youth of color received fewer days of outpatient therapy, potentially reflecting lower treatment uptake and higher premature dropout. Lower uptake and increased dropout among racially minoritized youth may be driven by negative youth/family experiences of treatment (e.g., poor cultural relevance of interventions, low cultural sensitivity of providers), increased structural/logistical barriers (e.g., lack of transportation), or mental health stigma in families and communities of color (Kapke & Gerdes, 2016; Planey, Smith, Moore, & Walker, 2019). Language barriers might also contribute to the especially pronounced disparities for Latinx youth, approximately two-thirds of whom indicated a preferred language other than English. Interestingly, however, preferred language was a less consistent predictor of treatment receipt than racial and ethnic identity, suggesting that broader cultural factors and provider/systems biases may be more impactful.

Results also revealed lower rates of psychotherapy receipt among youth with EDs relative to youth with other disorders. These findings are especially troubling because unlike mood/anxiety and psychotic disorders, which respond well to psychotropic medication, no medications are FDA-approved to treat EDs in youth (Golden & Attia, 2011). EDs have historically been misperceived as “diseases of affluence” that do not impact under-resourced youth (Burnette et al., 2024). Due in part to these historical misperceptions, providers within Medicaid-funded systems report low levels of training and confidence in treating EDs, contributing to high reliance on costly inpatient care (Accurso et al., 2025; Crest, Vendlinski, Borges, Landsverk, & Accurso, 2024; Mikhail et al., 2025). Indeed, although youth with psychotic disorders had the highest hospitalization rate in this study (which may reflect the fact that they often experience an emergency department visit or hospitalization leading up to diagnosis; Simon, Stewart, Hunkeler et al., 2018), youth with EDs had more hospitalizations for primarily medical treatment (see Supplementary Table S2) and significantly longer hospitalizations than youth with either mood/anxiety or psychotic disorders. They also received significantly more outpatient medical care. It is possible that lengthy hospitalizations and medical complications could be avoided for at least some youth with EDs if outpatient therapy was more readily accessible.

In addition to being less likely to receive outpatient therapy overall, youth with EDs generally experienced greater sex- and race and ethnicity-based treatment disparities than youth with mood/anxiety and psychotic disorders. Boys with EDs were less likely to receive any outpatient therapy and received fewer days of outpatient therapy than girls, while sex differences in outpatient therapy were absent or significantly attenuated for youth with mood/anxiety and psychotic disorders. Conversely, boys with EDs received more outpatient medical treatment than girls and had significantly longer hospitalizations when admitted, suggesting that the lack of outpatient therapy for boys may lead to a more severe and complicated course of illness. Similarly, while racial disparities were observed across the full sample, these were often amplified for youth with EDs, particularly those identifying as Latinx. Consequently, Latinx boys with EDs were only half as likely to receive any outpatient therapy as Latinx boys with mood/anxiety and psychotic disorders, while therapy receipt was more similar across diagnoses for White girls. Prior study suggests the full spectrum of EDs are present in publicly insured youth (Accurso et al., 2024), and sex- and race and ethnicity-based disparities in outpatient therapy persist for these with youth even after accounting for specific ED diagnosis (Mikhail et al., 2025; Moreno et al., 2023), indicating effects cannot be attributed to differences in the types of EDs affecting youth with different identity characteristics. Instead, they may reflect the fact ED research and treatment have rarely attended to low-income boys of color, who deviate from ED stereotypes across multiple aspects of identity, and increased stigma around care seeking for these youth. Current findings suggest that even when EDs are recognized in these youth, systemic biases, stigma, and/or difficulties engaging with treatments not designed with them in mind may negatively impact their care.

The current study had several strengths, including an intersectional lens, large sample size, use of state billing records to comprehensively identify all services received by youth within Medicaid, and actionable health policy implications. However, some limitations should be noted. We were unable to distinguish between the impact of lower rates of treatment referral (potentially reflecting systemic biases in treatment recommendations) and lower rates of referral utilization (potentially reflecting increased stigma or logistical barriers among certain youth/families) on service receipt. Additional research is required to understand the extent to which these factors may contribute to the patterns observed in this study.

While Medi-Cal is decentralized and therefore resembles state-to-state diversity in service environments, it is possible results may not fully generalize to youth in other states. Although we were able to capture all services within Medicaid, county behavioral health plans contract with private treatment centers when members require a higher level of care. These services are not captured in the current analyses because they cannot be billed to Medicaid. Data provided by the state were limited with respect to racial and ethnic identity (e.g., categories were mutually exclusive), which precluded our ability to identify multiracial participants (e.g., youth identifying as Black and Latinx) or distinguish between cultural groups within broad categories (e.g., Latinx participants from different countries of origin). Data also lacked a measure of disorder severity and only included sex rather than gender identity. To facilitate interpretability, ensure sufficient power, and avoid conducting an undue number of tests, we analyzed two-way interactions only rather than potential three-way effects. Our analyses may also underestimate the true disparities that exist among youth with these psychiatric diagnoses given that sex- and race and ethnicity-based biases impact their assessment and identification (Garb, 2021; Liang et al., 2016). Additional research, including qualitative research, is needed to fully understand the factors underlying the disparities observed.

The current findings have important implications for equitable health care for youth with mental illness. Across diagnoses and demographic factors, many youth received no outpatient therapy at all, and the number of sessions received was below that recommended in evidence-based protocols (Ehrenreich et al., 2009; Gorrell et al., 2019; Langman-Levy et al., 2022) or observed in other community settings (Lindstedt, Forss, Elwin, Kjellin, & Gustafsson, 2020; Weisz et al., 2009). Targeted efforts are important to ensure that all youth receive the care they need regardless of their demographic characteristics, with particular attention to youth who have been historically overlooked in research and practice (e.g., lower-income youth, youth of color, and boys with EDs). This may include efforts to eliminate referral biases or undue barriers to treatment for certain youth, reduce stigma in minoritized communities, and increase attention to cultural factors and building a strong therapeutic alliance, which can help ameliorate early therapy dropout among youth of color (de Haan, Boon, de Jong, & Vermeiren, 2018). Ultimately, ensuring adequate and equitable mental health treatment is critical to promote positive outcomes among socioeconomically vulnerable youth, which may also reduce treatment costs and costs to society.

## Supporting information

Accurso et al. supplementary material Accurso et al. supplementary material

## Supplementary material

The supplementary material for this article can be found at http://doi.org/10.1017/S003329172510069X.
