# Supplementary material for: A transdiagnostic examination of sex- and race and ethnicity-based mental health treatment disparities among publicly insured youth
Source: Psychol Med. 2025 Jun 24;55:e170. doi: 10.1017/S003329172510069X (PMC12201955; doi:10.1017/S003329172510069X)
Supplement: Accurso et al. supplementary material [file S003329172510069Xsup001.docx]

**Supplemental Material**

**Specific Disorders Included in Each Diagnostic Category**

**Eating disorders:** anorexia nervosa, bulimia nervosa, pica, rumination disorder, binge-eating disorder, avoidant/restrictive food intake disorder, other specified feeding/eating disorder, unspecified feeding/eating disorder.

**Mood/anxiety disorders:** bipolar I disorder without psychotic features, bipolar II disorder without psychotic features, cyclothymic disorder, other specified bipolar disorder, unspecified bipolar disorder, disruptive mood dysregulation disorder, major depressive disorder without psychotic features, persistent depressive disorder/dysthymic disorder, other specified depressive disorder, unspecified depressive disorder, separation anxiety disorder, specific phobia, social anxiety disorder/social phobia, panic disorder, agoraphobia, generalized anxiety disorder, anxiety disorder due to physiological condition, other anxiety disorder, unspecified anxiety disorder.

**Psychotic disorders:** bipolar I disorder with psychotic features, major depressive disorder with psychotic features, schizophrenia, schizoaffective disorder, brief psychotic disorder, schizophreniform disorder, other psychotic disorder, unspecified psychotic disorder.

**Identification of Youth in Each Diagnostic Group**

Diagnoses were derived from Medi-Cal billing records. Billing records examined in the current study included up to two diagnoses per visit. The sequencing of diagnoses (primary vs. secondary) can be influenced by administrative and billing priorities, rather than reflecting clinical severity or relevance (CMS, 2019), and youth with psychiatric conditions often meet criteria for multiple diagnoses. Therefore, incorporating secondary diagnoses is essential to ensure accurate case identification and comprehensive analysis in research. In this study, we considered both primary and secondary diagnoses to maximize sensitivity in identifying relevant youth in each diagnostic group.

Mood and anxiety disorders were considered together as a single group because they are highly comorbid across the lifespan (Saha et al., 2021), with particularly high comorbidity rates among adolescents (i.e., greater than 50%; Essau et al., 2018; Garber & Weersing, 2010; Melton et al., 2016). Empirical research further suggests that mood and anxiety disorders may be best understood as different expressions of a latent internalizing factor in children and adolescents (Wadsworth et al., 2001; Trosper et al., 2012). Consequently, treatments for anxiety disorders and depression in young people are very similar, with identical interventions often used to treat both types of disorders (e.g., selective serotonin reuptake inhibitors, CBT-based therapies such as the Unified Protocol; Ehrenreich et al., 2009; Murphy et al., 2021). Combining mood and anxiety disorders therefore made conceptual sense for a study of service use in young people, particularly in a setting where diagnoses are made by a range of providers (e.g., general practice pediatricians) who typically do not conduct formal diagnostic interviews that may be better able to distinguish between more nuanced mood and anxiety presentations.

**Classification of Youth with Diagnoses Across Multiple Categories**

For youth who had diagnoses across multiple categories, they were included in the eating disorder (ED) group if they had an ED (regardless of comorbid diagnoses) and in the psychotic disorder group if they had both a psychotic and mood/anxiety disorder but no ED. We assigned youth with EDs to the ED group even if additional diagnoses were present because comorbidities are extremely common in this population (i.e., over 50%, and in some cases up to 95%, of individuals with EDs in population-based samples meet criteria for at least one other psychiatric diagnosis, with rates of comorbidity likely higher in treatment-seeking samples; Hudson et al., 2007; Ulfvebrand et al., 2015). This approach also allowed for a more conservative test of our second aim examining whether treatment disparities were greater among youth with EDs than youth with other diagnoses. In other words, if youth with EDs (particularly those from minoritized backgrounds) received less care despite having a greater burden of psychiatric diagnoses overall, this would underscore the degree to which they are underserved. Similarly, psychotic disorders frequently present with comorbid mood/anxiety disorders (Wilson et al., 2020) and thus including youth with both psychotic disorders and mood/anxiety disorders in the psychotic disorder group made pragmatic and conceptual sense.

**Table S1.** *Descriptive statistics for participant demographics and service use in the second year after known diagnosis*

|  | **Eating Disorders (ED)** | | **Mood and Anxiety Disorders (MAD)** | | **Psychotic Disorders (PD)** | | **Group Comparison** |
| --- | --- | --- | --- | --- | --- | --- | --- |
| **Demographic Variables** | **N** | **%** | **N** | **%** | **N** | **%** |  |
| **All** | 1,293 | — | 1,378 | — | 1,150 | — | — |
|  |  |  |  |  |  |  |  |
| **Sex** |  |  |  |  |  |  |  |
| Female | 844 | 65.3 | 698 | 50.7 | 556 | 48.4 | MAD = PD < ED |
| Male | 449 | 34.7 | 680 | 49.4 | 594 | 51.7 | — |
|  |  |  |  |  |  |  |  |
| **Race and Ethnicity** |  |  |  |  |  |  |  |
| Asian/Pacific Islander | 50 | 3.9 | 47 | 3.4 | 33 | 2.9 | ED = MAD = PD |
| Black/African American | 47 | 3.6 | 99 | 7.2 | 97 | 8.4 | ED < MAD = PD |
| Latinx | 761 | 58.9 | 659 | 47.8 | 398 | 34.6 | PD < MAD < ED |
| Other/Unknown | 170 | 13.2 | 173 | 12.6 | 223 | 19.4 | ED = MAD < PD |
| White | 265 | 20.5 | 400 | 29.0 | 399 | 34.7 | ED < MAD < PD |
|  |  |  |  |  |  |  |  |
| **Language** |  |  |  |  |  |  |  |
| English | 630 | 48.7 | 864 | 62.7 | 799 | 69.5 | ED < MAD < PD |
| Other | 663 | 51.3 | 514 | 37.3 | 351 | 30.5 | — |
|  |  |  |  |  |  |  |  |
| **Age** | **Median** | **Mean (SD)** | **Median** | **Mean (SD)** | **Median** | **Mean (SD)** |  |
| At Known Diagnosis | 12.5 | 11.9 (2.6) | 12.2 | 11.8 (2.4) | 13.1 | 12.5 (2.3) | ED = MAD < PD |
|  |  |  |  |  |  |  |  |
| **Service Use** | **N (%)** | **Mean (SD)** | **N (%)** | **Mean (SD)** | **N (%)** | **Mean (SD)** |  |
| **Any Outpatient Therapy** | 303 (23.4) | 5.0 (6.0) | 451 (32.7) | 5.1 (7.0) | 553 (48.1) | 7.2 (9.6) | **N:** ED < MAD < PD  **Days:** ED = MAD < PD |
|  |  |  |  |  |  |  |  |
| *Family Therapy* | 240 (18.6) | 4.3 (5.6) | 382 (27.7) | 4.6 (6.7) | 530 (46.1) | 7.2 (9.7) | **N:** ED < MAD < PD  **Days:** ED = MAD < PD |
|  |  |  |  |  |  |  |  |
| *Individual Therapy* | 81 (6.3) | 6.0 (6.3) | 84 (6.1) | 6.3 (7.2) | 32 (2.8) | 5.3 (5.4) | **N:** PD < ED = MAD  **Days:** ED = MAD = PD |
|  |  |  |  |  |  |  |  |
| **Outpatient Medical Services** | 1,252 (96.8) | 15.2 (26.0) | 1,303 (94.6) | 8.8 (11.5) | 1,101 (95.7) | 11.6 (13.0) | **N:** MAD < ED, ED = PD, MAD = PD  **Days:** MAD < PD < ED |
|  |  |  |  |  |  |  |  |
| **Inpatient Admissions** | 117 (9.0) | 9.1 (11.9) | 100 (7.3) | 10.9 (37.8) | 181 (15.7) | 12.3 (20.4) | **N:** ED = MAD < PD  **Days:** ED = MAD = PD |
|  |  |  |  |  |  |  |  |
| *MH Admissions* | 81 (6.3) | 10.7 (13.3) | 65 (4.7) | 14.5 (46.3) | 161 (14.0) | 13.2 (21.3) | **N:** ED = MAD < PD  **Days:** ED = MAD = PD |
|  |  |  |  |  |  |  |  |
| *Medical Admissions* | 38 (2.9) | 5.1 (6.2) | 38 (2.8) | 3.7 (4.8) | 26 (2.3) | 3.4 (4.9) | **N:** ED = MAD = PD  **Days:** ED = MAD = PD |

*Note*. ED = eating disorder; MAD = mood/anxiety disorder; PD = psychotic disorder; MH = mental health; SD = standard deviation; N = number of participants receiving a given service; Days = number of days on which a service was received. Individual therapy was identified based on the following Current Procedural Terminology (CPT) codes: 90832–90834, 90836–90840, 90875, and 90876. Family therapy was identified using CPT codes 90846, 90847, and H0032. Means and standard deviations for service use categories are provided for participants who received at least one day of that service. Group comparisons were evaluated using chi-squared tests for categorical variables and t-tests for continuous variables at a significance level of *p* <.05. Dashes indicate that a value is not applicable (or is implied by a previous comparison).

**Table S2.** *Effects of demographic and diagnostic variables in predicting inpatient service use in first year after known diagnosis, distinguishing between primarily mental health and primarily medical admissions*

| **Models Without Interaction Effects** | | | | | |
| --- | --- | --- | --- | --- | --- |
|  |  | **Any MH Inpatient Admission** | **Days MH Inpatient Admission** | **Any Medical Inpatient Admission** | **Days Medical Inpatient Admission** |
|  |  | IRR (95% CI) | IRR (95% CI) | IRR (95% CI) | IRR (95% CI) |
|  |  |  |  |  |  |
| **Diagnostic Group (Ref = ED)** | |  |  |  |  |
| Mood/Anxiety Disorder (MAD) | | **.60 (.52, .69) ***** | **.45 (.39, .53) ***** | **.54 (.41, .71) ***** | **.25 (.16, .39) ***** |
| Psychotic Disorder (PD) | | **3.19 (2.92, 3.49) ***** | **.60 (.54, .67) ***** | **.43 (.32, .58) ***** | **.32 (.20, .51) ***** |
|  | |  |  |  |  |
| **Sex (Ref = Male)** | |  |  |  |  |
|  | Female | **1.44 (1.34, 1.54) ***** | 1.04 (.95, 1.14) | 1.18 (.92, 1.51) | .78 (.54, 1.14) |
|  | |  |  |  |  |
| **Race and Ethnicity (Ref = White)** | |  |  |  |  |
| Asian/Pacific Islander | | .91 (.76, 1.08) | .87 (.70, 1.08) | .69 (.35, 1.37) | **7.09 (2.57, 19.53) ***** |
|  | Black | **.75 (.65, .88) ***** | **.75 (.63, .91) **** | 1.42 (.91, 2.23) | **3.24 (1.63, 6.41) **** |
|  | Latinx | **.83 (.77, .90) ***** | **.69 (.62, .75) ***** | **.73 (.55, .98)** * | **1.66 (1.06, 2.60) *** |
|  | Other/Unknown | **.85 (.77, .94) **** | **1.29 (1.13, 1.46) ***** | **1.95 (1.43, 2.66) ***** | **6.04 (3.69, 9.89) ***** |
|  |  |  |  |  |  |
| **Age at First Known Diagnosis** | | **1.13 (1.11, 1.14) ***** | **.95 (.93, .97) ***** | **1.05 (1.00, 1.10) *** | **.89 (.85, .94) ***** |
| **Models with Interaction Effects** | | | | | |
|  | | **Any MH Inpatient Admission** | **Days MH Inpatient Admission** | **Any Medical Inpatient Admission** | **Days Medical Inpatient Admission** |
|  | | IRR (95% CI) | IRR (95% CI) | IRR (95% CI) | IRR (95% CI) |
|  |  |  |  |  |  |
| **Diagnostic Group (Ref = ED)** | |  |  |  |  |
| MAD | | **.60 (.42, .86) **** | **.32 (.22, .47) ***** | — | — |
| PD | | **3.70 (2.88, 4.76) ***** | **.46 (.34, .62) ***** | — | — |
|  | |  |  |  |  |
| **Sex (Ref = Male)** | |  |  |  |  |
|  | Female | **2.25 (1.77, 2.86) ***** | **.66 (.52, .86) **** | — | — |
|  | |  |  |  |  |
| **Race and Ethnicity (Ref = White)** | |  |  |  |  |
| Asian/Pacific Islander | | .72 (.51, 1.03) | 1.05 (.71, 1.54) | — | — |
|  | Black | .73 (.49, 1.10) | **.45 (.28, .71) **** | — | — |
|  | Latinx | **.53 (.45, .64) ***** | **.68 (.56, .83) ***** | — | — |
|  | Other/Unknown | **.77 (.60, .99) *** | **2.23 (1.69, 2.95) ***** | — | — |
|  | |  |  |  |  |
| **Age at First Known Diagnosis** | | **1.12 (1.11, 1.14) ***** | **.96 (.94, .98) ***** | — | — |
|  | |  |  |  |  |
| **Model Interaction Terms** | |  |  |  |  |
|  | |  |  |  |  |
| **Diagnosis x Sex** | |  |  |  |  |
| MAD x sex | | .74 (.53, 1.04) | **1.50 (1.04, 2.17) *** | — | — |
| PD x sex | | **.57 (.44, .73) ***** | **1.71 (1.31, 2.25) ***** | — | — |
|  | |  |  |  |  |
| **Diagnosis x Race and Ethnicity** | |  |  |  |  |
| MAD x Asian/Pacific Islander | | 1.20 (.60, 2.39) | .75 (.35, 1.58) | — | — |
| MAD x Black | | 1.07 (.56, 2.04) | 1.92 (.96, 3.87) | — | — |
| MAD x Latinx | | **1.51 (1.10, 2.07) *** | 1.11 (.79, 1.57) | — | — |
| MAD x Other/Unknown | | 1.52 (.98, 2.36) | .82 (.51, 1.31) | — | — |
|  | |  |  |  |  |
| PD x Asian/Pacific Islander | | 1.34 (.89, 2.01) | .77 (.48, 1.25) | — | — |
| PD x Black | | 1.05 (.68, 1.64) | **1.77 (1.06, 2.93) *** | — | — |
| PD x Latinx | | **1.84 (1.51, 2.23) ***** | .97 (.77, 1.23) | — | — |
| PD x Other/Unknown | | 1.09 (.83, 1.43) | **.41 (.30, .57) ***** | — | — |

*Note*. ED = eating disorder; MAD = mood/anxiety disorder; PD = psychotic disorder; MH = mental health; IRR = incidence rate ratio. The number of service days was analyzed for participants who received at least one instance of a given service type. Inpatient days that were split between mental health and medical services were included in both types of analyses. Interaction effects were not computed for medical admissions because the total number of participants with a primarily medical hospitalization was relatively small (*n* = 297). ** <.05; ** <.01; *** <.001*

**Table S3.** *Effects of demographic and diagnostic variables in predicting service receipt in the first year after known diagnosis, covarying other service types*

| **Models Without Interaction Effects** | | | | | | |
| --- | --- | --- | --- | --- | --- | --- |
|  |  | **Any Outpatient**  **Therapy Services** | **Days Outpatient Therapy Services** | **Days Outpatient Medical Services** | **Any Inpatient Admission** | **Days Inpatient** |
|  |  | IRR (95% CI) | IRR (95% CI) | IRR (95% CI) | IRR (95% CI) | IRR (95% CI) |
|  |  |  |  |  |  |  |
| **Diagnostic Group (Ref = ED)** | |  |  |  |  |  |
| Mood/Anxiety Disorder (MAD) | | **1.34 (1.27, 1.41) ***** | .94 (.87, 1.01) | **.62 (.60, .65) ***** | **.61 (.54, .69) ***** | **.53 (.46, .61) ***** |
| Psychotic Disorder (PD) | | **1.33 (1.26, 1.41) ***** | **1.09 (1.01, 1.18) *** | **.77 (.73, .81) ***** | **2.42 (2.23, 2.62) ***** | **.70 (.64, .78) ***** |
|  | |  |  |  |  |  |
| **Sex (Ref = Male)** | |  |  |  |  |  |
|  | Female | **1.05 (1.01, 1.10) *** | .99 (.93, 1.05) | **.89 (.85, .92)** *** | **1.38 (1.29, 1.47) ***** | 1.03 (.94, 1.12) |
|  | |  |  |  |  |  |
| **Race and Ethnicity (Ref = White)** | |  |  |  |  |  |
| Asian/Pacific Islander | | .97 (.87, 1.08) | **.83 (.71, .96) *** | **.84 (.76, .93) **** | .94 (.79, 1.11) | 1.13 (.92, 1.39) |
|  | Black | .97 (.89, 1.05) | **.84 (.74, .94) **** | **.87 (.80, .95) **** | **.86 (.75, .99) *** | .89 (.75, 1.06) |
|  | Latinx | **.91 (.87, .95) ***** | **.83 (.78, .89) ***** | **.78 (.74, .81)** *** | **.89 (.83, .96) **** | **.79 (.72, .87) ***** |
|  | Other/Unknown | .98 (.92, 1.04) | **.89 (.82, .98) *** | 1.06 (1.00, 1.13) | .97 (.89, 1.07) | **1.31 (1.16, 1.49) ***** |
|  |  |  |  |  |  |  |
| **Age at First Known Diagnosis** | | 1.00 (1.00, 1.01) | **.98 (.97, .99) ***** | **.96 (.95, .97) ***** | **1.12 (1.11, 1.13) ***** | .99 (.97, 1.00) |
|  | |  |  |  |  |  |
| **Other Service Types** | |  |  |  |  |  |
| Any Outpatient Therapy Services | | **—** | **—** | **.88 (.84, .92) ***** | **1.40 (1.30, 1.50) ***** | 1.03 (.94, 1.13) |
| Days Outpatient Therapy Services | | **—** | **—** | 1.00 (1.00, 1.00) | **1.02 (1.01, 1.02) ***** | **1.01 (1.01, 1.02) ***** |
| Days Outpatient Medical Services | | **.96 (.95, .98) ***** | 1.00 (.97, 1.02) | **—** | **1.10 (1.08, 1.11) ***** | **1.30 (1.27, 1.34) ***** |
| Any Inpatient Admission | | **1.42 (1.35, 1.49) ***** | **1.37 (1.27, 1.48) ***** | **1.28 (1.22, 1.35) ***** | **—** | **—** |
| Days Inpatient | | **.99 (.98, .997) *** | **1.08 (1.05, 1.11) ***** | **1.06 (1.05, 1.07) ***** | **—** | **—** |
| **Models with Interaction Effects** | | | | | | |
|  | | **Any Outpatient**  **Therapy Services** | **Days Outpatient Therapy Services** | **Days Outpatient Medical Services** | **Any Inpatient Admission** | **Days Inpatient** |
|  | | IRR (95% CI) | IRR (95% CI) | IRR (95% CI) | IRR (95% CI) | IRR (95% CI) |
|  |  |  |  |  |  |  |
| **Diagnostic Group (Ref = ED)** | |  |  |  |  |  |
| MAD | | **1.55 (1.35, 1.78) ***** | 1.00 (.84, 1.19) | **.51 (.45, .56) ***** | **.54 (.40, .72) ***** | **.49 (.36, .68) ***** |
| PD | | **1.50 (1.31, 1.71) ***** | **1.28 (1.08, 1.51) **** | **.66 (.60, .74) ***** | **2.39 (1.95, 2.93) ***** | **.71 (.55, .91) **** |
|  | |  |  |  |  |  |
| **Sex (Ref = Male)** | |  |  |  |  |  |
|  | Female | **1.32 (1.19, 1.46) ***** | 1.10 (.97, 1.25) | **.74 (.69, .79)** *** | **1.73 (1.44, 2.07) ***** | **.78 (.64, .96) *** |
|  | |  |  |  |  |  |
| **Race and Ethnicity (Ref = White)** | |  |  |  |  |  |
| Asian/Pacific Islander | | 1.00 (.83, 1.21) | .91 (.71, 1.17) | **.81 (.69, .95) **** | **.72 (.52, .99) *** | **1.74 (1.21, 2.50) **** |
|  | Black | .79 (.62, 1.02) | .99 (.73, 1.34) | .96 (.81, 1.15) | .85 (.60, 1.20) | 1.34 (.90, 1.98) |
|  | Latinx | **.84 (.76, .93) **** | **.85 (.75, .97) *** | **.76 (.71, .83)** *** | **.60 (.51, .70) ***** | .86 (.72, 1.03) |
|  | Other/Unknown | .94 (.82, 1.09) | .84 (.70, 1.01) | 1.06 (.95, 1.18) | .98 (.81, 1.19) | **2.75 (2.16, 3.51) ***** |
|  | |  |  |  |  |  |
| **Age at First Known Diagnosis** | | 1.00 (1.00, 1.01) | **.98 (.97, .99) ***** | **.96 (.96, .97) ***** | **1.12 (1.10, 1.13) ***** | .99 (.97, 1.01) |
|  | |  |  |  |  |  |
| **Model Interaction Terms** | |  |  |  |  |  |
|  | |  |  |  |  |  |
| **Diagnosis x Sex** | |  |  |  |  |  |
| MAD x sex | | **.69 (.61, .78) ***** | .92 (.79, 1.08) | **1.32 (1.20, 1.45) ***** | .88 (.67, 1.16) | **1.44 (1.05, 1.96) *** |
| PD x sex | | **.80 (.71, .90) ***** | **.84 (.71, .98) *** | **1.30 (1.18, 1.43) ***** | **.73 (.60, .89) **** | **1.41 (1.12, 1.77) **** |
|  | |  |  |  |  |  |
| **Diagnosis x Race and Ethnicity** | |  |  |  |  |  |
| MAD x Asian/Pacific Islander | | .96 (.73, 1.25) | .87 (.60, 1.25) | 1.09 (.86, 1.39) | 1.37 (.74, 2.52) | .52 (.26, 1.02) |
| MAD x Black | | **1.44 (1.09, 1.91) *** | .84 (.59, 1.21) | .83 (.67, 1.04) | 1.06 (.62, 1.83) | .67 (.37, 1.23) |
| MAD x Latinx | | **1.16 (1.02, 1.31) *** | 1.01 (.85, 1.19) | 1.05 (.94, 1.18) | **1.42 (1.07, 1.89) *** | .91 (.66, 1.25) |
| MAD x Other/Unknown | | 1.03 (.86, 1.24) | 1.13 (.88, 1.44) | 1.12 (.95, 1.31) | **1.53 (1.08, 2.17) *** | **.48 (.32, .71) ***** |
|  | |  |  |  |  |  |
| PD x Asian/Pacific Islander | | .93 (.72, 1.19) | .87 (.60, 1.25) | 1.00 (.77, 1.28) | 1.44 (.99, 2.11) | **.54 (.34, .86) **** |
| PD x Black | | 1.17 (.88, 1.54) | .80 (.56, 1.15) | .91 (.73, 1.13) | 1.02 (.70, 1.49) | **.61 (.39, .95) *** |
| PD x Latinx | | 1.08 (.96, 1.22) | .94 (.80, 1.11) | .99 (.89, 1.11) | **1.78 (1.50, 2.12) ***** | .88 (.71, 1.09) |
| PD x Other/Unknown | | 1.06 (.90, 1.25) | 1.05 (.84, 1.31) | .93 (.81, 1.08) | .90 (.72, 1.13) | **.33 (.24, .43) ***** |
|  | |  |  |  |  |  |
| **Other Service Types** | |  |  |  |  |  |
| Any Outpatient Therapy Services | | **—** | **—** | **.89 (.85, .93) ***** | **1.39 (1.29, 1.49) ***** | 1.09 (.99, 1.20) |
| Days Outpatient Therapy Services | | **—** | **—** | 1.00 (1.00, 1.00) | **1.02 (1.01, 1.02) ***** | **1.01 (1.01, 1.02) ***** |
| Days Outpatient Medical Services | | **.97 (.95, .98) ***** | 1.00 (.98, 1.02) | **—** | **1.10 (1.08, 1.11) ***** | **1.26 (1.23, 1.29) ***** |
| Any Inpatient Admission | | **1.41 (1.34, 1.48) ***** | **1.37 (1.27, 1.48) ***** | **1.27 (1.21, 1.33) ***** | **—** | **—** |
| Days Inpatient | | **.99 (.98, .997) *** | **1.08 (1.04, 1.11) ***** | **1.06 (1.05, 1.07) ***** | **—** | **—** |

*Note*. ED = eating disorder; MAD = mood/anxiety disorder; PD = psychotic disorder; IRR = incidence rate ratio. The number of service days was analyzed for participants who received at least one instance of a given service type. Receipt of any outpatient medical services was not examined as an outcome because almost all youth received at least some outpatient medical care. Number of days of outpatient medical services and number of days of inpatient care were divided by 10 in analyses where they were included as covariates to increase the interpretability of coefficients. ** <.05; ** <.01; *** <.001*

**Table S4.** *Effects of demographic and diagnostic variables in predicting service use in second year after known diagnosis*

|  |  | **Any Outpatient**  **Therapy Services** | **Days Outpatient Therapy Services** | **Days Outpatient Medical Services** | **Any Inpatient Admission** | **Days Inpatient** |
| --- | --- | --- | --- | --- | --- | --- |
|  |  | IRR (95% CI) | IRR (95% CI) | IRR (95% CI) | IRR (95% CI) | IRR (95% CI) |
|  |  |  |  |  |  |  |
| **Diagnostic Group (Ref = ED)** | |  |  |  |  |  |
| Mood/Anxiety Disorder (MAD) | | **1.36 (1.20, 1.54) ***** | .96 (.82, 1.12) | **.58 (.53, .62) ***** | .83 (.65, 1.07) | 1.00 (.75, 1.34) |
| Psychotic Disorder (PD) | | **1.91 (1.70, 2.16) ***** | **1.30 (1.12, 1.51) ***** | **.75 (.69, .82) ***** | **1.56 (1.25, 1.94) ***** | 1.26 (.96, 1.65) |
|  | |  |  |  |  |  |
| **Sex (Ref = Male)** | |  |  |  |  |  |
|  | Female | 1.04 (.95, 1.13) | .91 (.80, 1.02) | **.89 (.83, .95) ***** | **1.62 (1.33, 1.98) ***** | 1.20 (.95, 1.53) |
|  | |  |  |  |  |  |
| **Race and Ethnicity (Ref = White)** | |  |  |  |  |  |
| Asian/Pacific Islander | | .80 (.62, 1.03) | .76 (.55, 1.05) | 1.12 (.94, 1.35) | .66 (.38, 1.14) | .85 (.46, 1.57) |
| Black/African American | | .86 (.72, 1.02) | .80 (.64, 1.01) | .93 (.81, 1.08) | .91 (.63, 1.31) | **.48 (.31, .73) **** |
| Latinx | | **.71 (.64, .78) ***** | **.65 (.57, .74) ***** | **.77 (.71, .83) ***** | **.56 (.45, .69) ***** | **.64 (.50, .83) **** |
| Other/Unknown | | .94 (.83, 1.06) | **.74 (.63, .87) ***** | 1.10 (.99, 1.22) | 1.06 (.82, 1.37) | .99 (.73, 1.33) |
|  |  |  |  |  |  |  |
| **Age at First Known Diagnosis** | | 1.00 (.99, 1.02) | 1.01 (.99, 1.04) | **.96 (.95, .98) ***** | **1.14 (1.09, 1.19) ***** | **.91 (.87, .96) **** |

*Note*. ED = eating disorder; MAD = mood/anxiety disorder; PD = psychotic disorder; IRR = incidence rate ratio. The number of service days was analyzed for participants who received at least one instance of a given service type. Receipt of any outpatient medical services was not examined as an outcome because almost all youth received at least some outpatient medical care. Interaction effects with diagnosis were not examined for the second year of services due to smaller sample sizes. ** <.05; ** <.01; *** <.001*

**Table S5.** *Effects of demographic and diagnostic variables in predicting service use in first year after known diagnosis with language as a predictor in the model*

| **Models Without Interaction Effects** | | | | | | |
| --- | --- | --- | --- | --- | --- | --- |
|  |  | **Any Outpatient**  **Therapy Services** | **Days Outpatient Therapy Services** | **Days Outpatient Medical Services** | **Any Inpatient Admission** | **Days Inpatient** |
|  |  | IRR (95% CI) | IRR (95% CI) | IRR (95% CI) | IRR (95% CI) | IRR (95% CI) |
|  |  |  |  |  |  |  |
| **Diagnostic Group (Ref = ED)** | |  |  |  |  |  |
| Mood/Anxiety Disorder (MAD) | | **1.35 (1.27, 1.42) ***** | **.89 (.82, .96) **** | **.58 (.55, .60) ***** | **.58 (.51, .65) ***** | **.31 (.27, .36) ***** |
| Psychotic Disorder (PD) | | **1.52 (1.44, 1.61) ***** | **1.31 (1.21, 1.40) ***** | **.81 (.77, .85) ***** | **2.55 (2.35, 2.76) ***** | **.42 (.38, .46) ***** |
|  | |  |  |  |  |  |
| **Sex (Ref = Male)** | |  |  |  |  |  |
|  | Female | **1.09 (1.04, 1.13) ***** | 1.01 (.95, 1.08) | **.89 (.86, .93)** *** | **1.39 (1.30, 1.49) ***** | .94 (.85, 1.03) |
|  | |  |  |  |  |  |
| **Language (Ref = English)** | |  |  |  |  |  |
| Other | | .97 (.93, 1.01) | 1.00 (.94, 1.06) | **.93 (.89, .97) ***** | **.85 (.79, .91) ***** | .97 (.88, 1.07) |
|  |  |  |  |  |  |  |
| **Age at First Known Diagnosis** | | **1.01 (1.01, 1.02) ***** | **.98 (.97, .995) **** | **.97 (.96, .97) ***** | **1.12 (1.10, 1.13) ***** | **.93 (.91, .94) ***** |
| **Models with Interaction Effects** | | | | | | |
|  | | **Any Outpatient**  **Therapy Services** | **Days Outpatient Therapy Services** | **Days Outpatient Medical Services** | **Any Inpatient Admission** | **Days Inpatient** |
|  | | IRR (95% CI) | IRR (95% CI) | IRR (95% CI) | IRR (95% CI) | IRR (95% CI) |
|  |  |  |  |  |  |  |
| **Diagnostic Group (Ref = ED)** | |  |  |  |  |  |
| MAD | | **1.70 (1.51, 1.90) ***** | .94 (.81, 1.08) | **.47 (.43, .51) ***** | **.63 (.49, .81) ***** | **.17 (.13, .24) ***** |
| PD | | **1.70 (1.52, 1.90) ***** | **1.39 (1.20, 1.60) ***** | **.66 (.60, .71) ***** | **2.61 (2.16, 3.15) ***** | **.25 (.20, .32) ***** |
|  | |  |  |  |  |  |
| **Sex (Ref = Male)** | |  |  |  |  |  |
|  | Female | **1.38 (1.25, 1.54) ***** | **1.16 (1.02, 1.32) *** | **.75 (.70, .80)** *** | **1.71 (1.42, 2.07) ***** | **.52 (.42, .65) ***** |
|  | |  |  |  |  |  |
| **Language (Ref = English)** | |  |  |  |  |  |
| Other | | **.90 (.82, .98) *** | .90 (.80, 1.00) | **.87 (.82, .93) ***** | **.62 (.53, .71) ***** | 1.02 (.85, 1.22) |
|  | |  |  |  |  |  |
| **Age at First Known Diagnosis** | | **1.01 (1.01, 1.02) **** | **.98 (.97, .99) **** | **.97 (.96, .97) ***** | **1.11 (1.10, 1.13) ***** | **.93 (.91, .95) ***** |
|  | |  |  |  |  |  |
| **Model Interaction Terms** | |  |  |  |  |  |
|  | |  |  |  |  |  |
| **Diagnosis x Sex** | |  |  |  |  |  |
| MAD x sex | | **.67 (.59, .75) ***** | .86 (.74, 1.02) | **1.28 (1.16, 1.41) ***** | .84 (.63, 1.12) | **2.33 (1.66, 3.26) ***** |
| PD x sex | | **.79 (.70, .90) ***** | **.83 (.71, .97) *** | **1.32 (1.19, 1.45) ***** | **.76 (.62, .93) **** | **2.08 (1.62, 2.67) ***** |
|  | |  |  |  |  |  |
| **Diagnosis x Language** | |  |  |  |  |  |
| MAD x other | | 1.08 (.96, 1.20) | 1.13 (.98, 1.32) | **1.12 (1.02, 1.24) *** | 1.09 (.84, 1.43) | .86 (.62, 1.19) |
| PD x other | | **1.18 (1.06, 1.31) **** | **1.20 (1.04, 1.40) *** | 1.09 (.99, 1.21) | **1.68 (1.43, 1.98) ***** | .90 (.73, 1.12) |

*Note*. ED = eating disorder; MAD = mood/anxiety disorder; PD = psychotic disorder; IRR = incidence rate ratio. The number of service days was analyzed for participants who received at least one instance of a given service type. Receipt of any outpatient medical services was not examined as an outcome because almost all youth received at least some outpatient medical care. ** <.05; ** <.01; *** <.001*

*
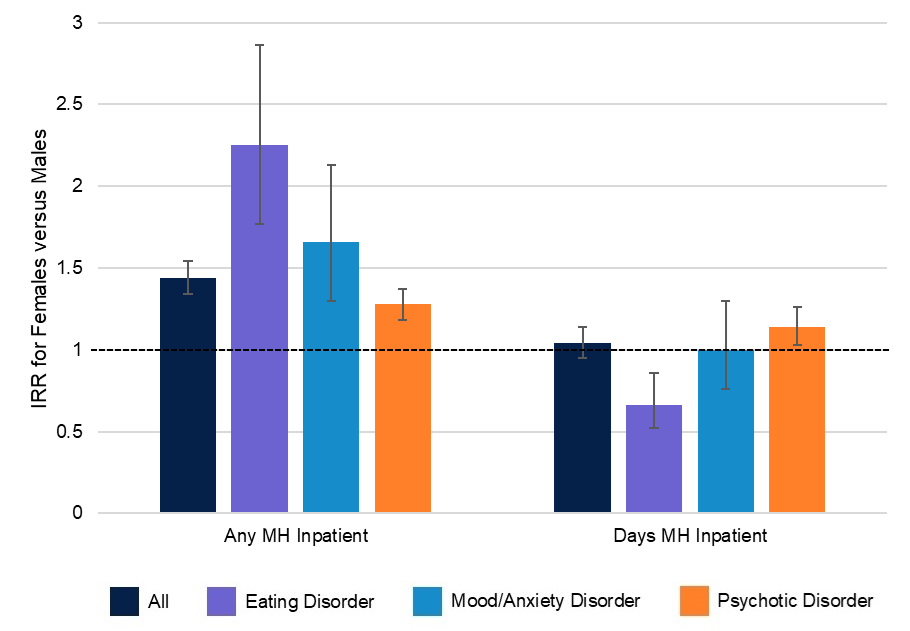
*

**Figure S1.** *Sex differences in* *mental health inpatient admissions by diagnosis.* MH = mental health; IRR = incidence rate ratio. Error bars represent 95% confidence intervals and the dotted line represents an IRR of 1 (indicating no difference between females and males).

**
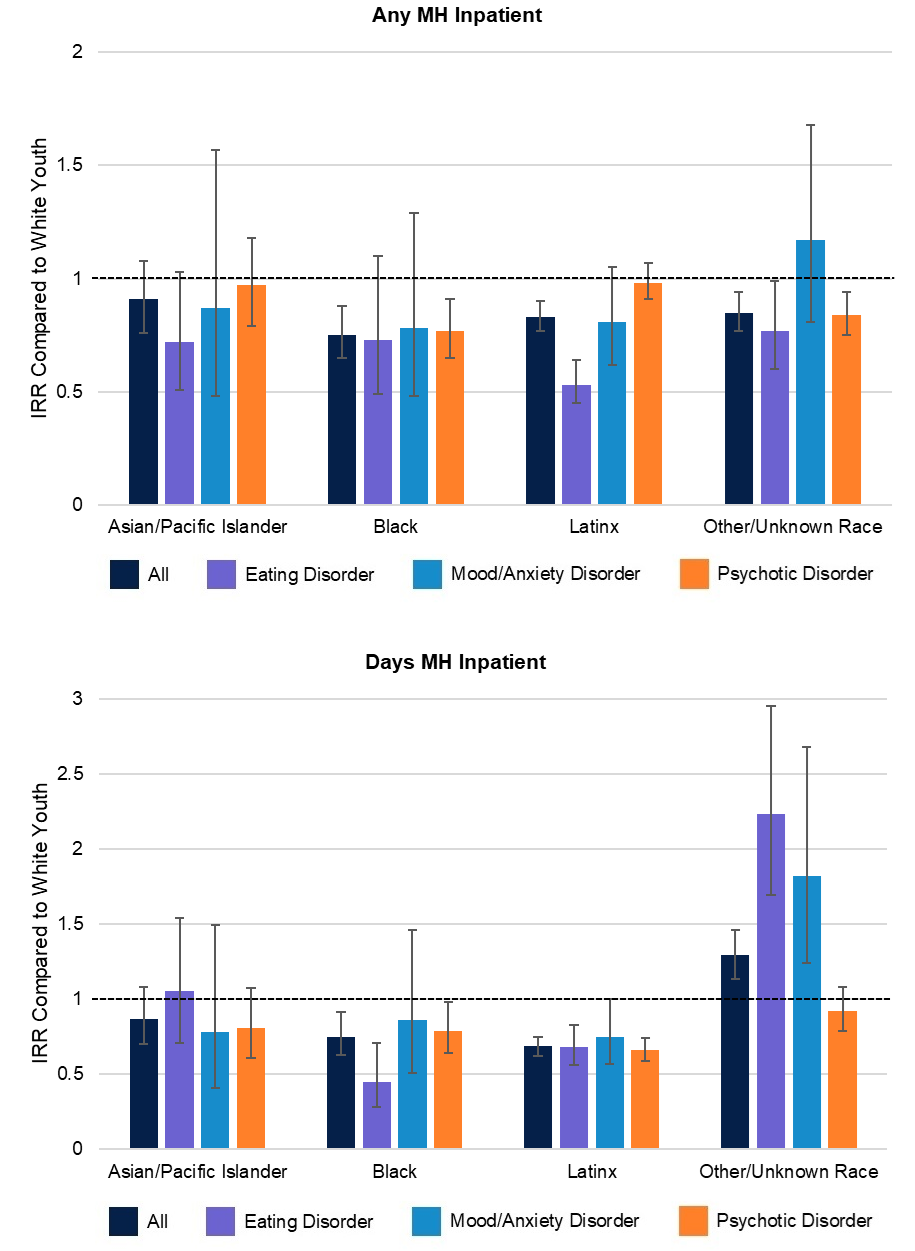
**

**Figure S2.** *Racial and ethnic differences in* *mental health inpatient admissions by diagnosis.* MH = mental health; IRR = incidence rate ratio. Error bars represent 95% confidence intervals and the dotted line represents an IRR of 1 (indicating no difference with White youth).

**
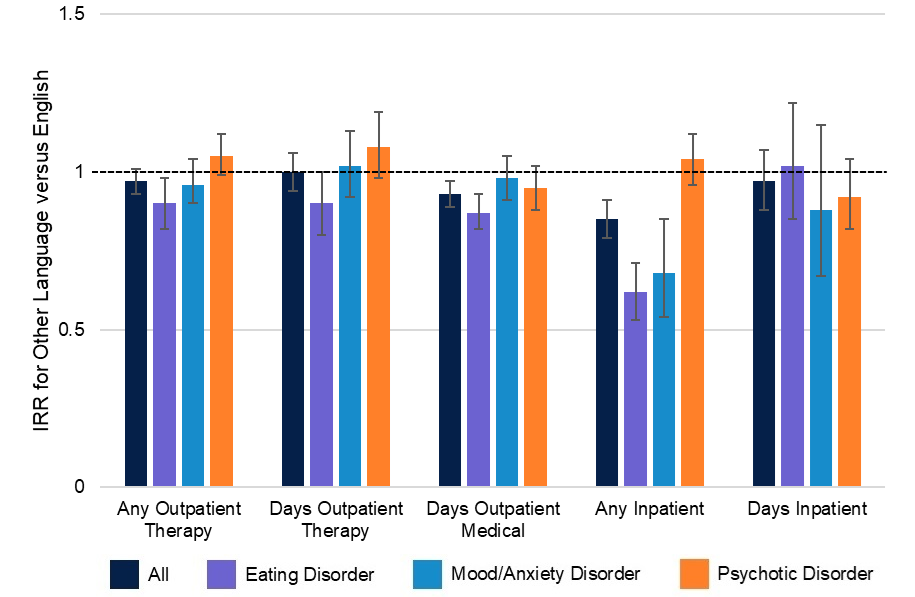
**

**Figure S3.** *Differences in services received by preferred language.* IRR = incidence rate ratio. Error bars represent 95% confidence intervals and the dotted line represents an IRR of 1 (indicating no difference between youth with a preferred language other than English and youth with English as a preferred language).

**References**

Centers for Medicare and Medicaid Services. (2019). *ICD-10-CM Official Guidelines for Coding and Reporting FY 2019*. <https://archive.cdc.gov/www_cdc_gov/nchs/icd/data/10cmguidelines-FY2019-final.pdf>

Ehrenreich, J. T., Goldstein, C. R., Wright, L. R., & Barlow, D. H. (2009). Development of a

unified protocol for the treatment of emotional disorders in youth. *Child & Family Behavior Therapy*, *31*(1), 20-37.

Essau, C. A., Lewinsohn, P. M., Lim, J. X., Ho, M. H. R., & Rohde, P. (2018). Incidence,

recurrence and comorbidity of anxiety disorders in four major developmental stages. *Journal of Affective Disorders*, *228*, 248-253.

Garber, J., & Weersing, V. R. (2010). Comorbidity of anxiety and depression in youth:

Implications for treatment and prevention. *Clinical psychology: Science and Practice*, *17*(4), 293.

Melton, T. H., Croarkin, P. E., Strawn, J. R., & Mcclintock, S. M. (2016). Comorbid anxiety and

depressive symptoms in children and adolescents: A systematic review and analysis. *Journal of Psychiatric Practice*, *22*(2), 84-98.

Murphy, S. E., Capitão, L. P., Giles, S. L., Cowen, P. J., Stringaris, A., & Harmer, C. J. (2021).

The knowns and unknowns of SSRI treatment in young people with depression and anxiety: Efficacy, predictors, and mechanisms of action. *The Lancet Psychiatry*, *8*(9), 824-835.

Saha, S., Lim, C. C., Cannon, D. L., Burton, L., Bremner, M., Cosgrove, P.,...J McGrath, J.

(2021). Co‐morbidity between mood and anxiety disorders: A systematic review and meta‐analysis. *Depression and Anxiety*, *38*(3), 286-306.

Trosper, S. E., Whitton, S. W., Brown, T. A., & Pincus, D. B. (2012). Understanding the latent

structure of the emotional disorders in children and adolescents. *Journal of Abnormal Child Psychology*, *40*, 621-632.

Ulfvebrand, S., Birgegård, A., Norring, C., Högdahl, L., & von Hausswolff-Juhlin, Y. (2015).

Psychiatric comorbidity in women and men with eating disorders: Results from a large clinical database. *Psychiatry Research*, *230*(2), 294-299.

Wadsworth, M. E., Hudziak, J. J., Heath, A. C., & Achenbach, T. M. (2001). Latent class analysis of child behavior checklist anxiety/depression in children and adolescents. *Journal of the American Academy of Child & Adolescent Psychiatry*, *40*(1), 106-114.

Wilson, R. S., Yung, A. R., & Morrison, A. P. (2020). Comorbidity rates of depression and

anxiety in first episode psychosis: A systematic review and meta-analysis. *Schizophrenia Research*, *216*, 322-329.
